# Sealing the Breach: A Surgical Solution for Tracheoesophageal Fistula With a Simple Two-Layer Closure

**DOI:** 10.7759/cureus.61934

**Published:** 2024-06-08

**Authors:** Siti Hajar Noordiana, Florence Tan Hui Fang, Nur Asyiqin Kamarudin, Mawaddah Azman

**Affiliations:** 1 Department of Otorhinolaryngology - Head and Neck Surgery, Universiti Kebangsaan Malaysia Medical Centre, Kuala Lumpur, MYS; 2 Department of Otorhinolaryngology, Hospital Selayang, Selayang, MYS; 3 Department of Otolaryngology - Head and Neck Surgery, Universiti Kebangsaan Malaysia Medical Centre, Kuala Lumpur, MYS

**Keywords:** tracheoesophageal puncture, aspiration pneumonia, fistula closure, speech prosthesis, leakage, laryngectomy complications, voice rehabilitation, prosthetic speech, tracheoesophageal fistula

## Abstract

Tracheoesophageal puncture (TEP) followed by voice prosthesis placement stands as the primary method for voice rehabilitation after laryngectomy, heralded for its effectiveness. While generally well-tolerated, the procedure does pose potential long-term complications. These include prosthesis valve leakage, scarring, and prosthesis displacement, all of which can impede phonation capabilities. Of these, prosthesis leakage emerges as the most critical concern, precipitated by the progressive widening of the fistula. This complication can precipitate aspiration pneumonitis, stemming from the loss of physical separation between the esophagus and trachea. This case series details three instances where persistent tracheoesophageal fistula arose following TEP, necessitating surgical intervention. Herein, we present the clinical manifestations, surgical approach employing a simple two-layer closure, and ensuing outcomes.

## Introduction

Laryngeal and pyriform fossa cancers comprise 8-9% of all head and neck cancers. Stages 3 and 4 require total laryngectomy along with postoperative radiation therapy [1]. Total laryngectomy poses a significant challenge as it deprives individuals of speech, greatly impacting their ability to communicate and profoundly affecting their social, psychological, and economic well-being. The restoration of speech in these patients is paramount for enhancing their quality of life. While esophageal voice serves as an alternative for laryngectomees, its adoption requires extensive and prolonged speech therapy. Unfortunately, the acquisition rate for esophageal voice remains relatively low, standing at only 30% [2].

Advancements in voice rehabilitation led to the introduction of surgically created tracheoesophageal puncture (TEP) and the fitting of these fistulas with voice prostheses. Innovations in indwelling prostheses over time led to a variety of available prostheses which vary regarding their frequency of complications, in situ lifetime, and the quality of voice produced [3].

The complications include leakage due to valve failure, scarring and granulation, tracheostomal stenosis, cervical cellulitis, and prosthesis displacement [4]. Usually, these complications can be treated by either replacing the prosthesis or, in the case of fistula, by temporary removal of the prosthesis. However, in certain cases, the fistula persists despite these actions. As a result, they have to be closed surgically to prevent potentially life-threatening complications such as aspiration pneumonia [3].

This case series details three instances where persistent tracheoesophageal fistula (TEF) arose following TEP, necessitating surgical intervention. Herein, we present the clinical manifestations, surgical approach employing a simple two-layer closure, and ensuing outcomes.

## Case presentation

Case 1

The first case is of a 78-year-old Indian gentleman with underlying hypertension, ischemic heart disease, and gouty arthritis. The patient had a history of recurrent glottic carcinoma post radiotherapy (rT2N0M0), for which he had undergone a total laryngectomy followed by a TEP. The patient was using a tracheoesophageal voice prosthesis for 11 years until he noticed he was no longer able to talk using his voice prosthesis. However, no aspiration symptoms on fluids or solids were noted. The patient otherwise had no difficulty breathing, no dysphagia, no odynophagia, and was able to feed orally well. On physical examination, stoma stenosis was seen, and the voice prosthesis had migrated externally. No leaking was observed upon taking fluids. However, when the stoma was occluded for voicing, the tracheal lumen of the prosthesis was persistently occluded by the finger, preventing air entry. 

A flexible endoscopy showed normal neopharynx and tracheal mucosa. The migrated prosthesis was removed in view of a non-functioning TEP, and the patient was put on exclusive nasogastric tube feeding for one month. The patient was later planned for repair of TEF under local anesthesia in view of a persistent fistula. Intraoperatively, a small TEF was seen at 1 o’clock, superior to the mucocutaneous junction of the tracheal stoma. An elliptical incision was made surrounding the fistula, and a subplatysmal skin flap was raised. The serosal wall of the esophagus and the fistula tract were delineated with sharp dissection. Subsequently, a cuff of mucosa surrounding the tracheal and the esophageal ends of the fistula was removed and refashioned. The esophageal mucosa was repaired vertically, followed by another layer of repair at the serosal layer using polyglactin 3-0 sutures. The tracheal defect was repaired horizontally using polyglactin 3-0 sutures, followed by skin flap closure. Postoperative care included prophylactic antibiotics (oral amoxycillin clavulanate 15 mg/kg/dose BD) for three days, and analgesia (oral paracetamol 10 mg/kg/dose TDS), sterile water dressing, and exclusive nasogastric tube feeding for two weeks. At two weeks clinic visit, evidence of healing was noted, and no leak was noted on the clear fluid swallowing test. The nasogastric tube was removed, and the patient was instructed to resume feeding perorally. No intermediate complications were noted, and the patient started to use the electrolarynx for voice rehabilitation two months after the closure of the TEF. Long-term follow-up at one year post-procedure showed good healing and no recurrence of TEF.

Case 2

The second case was of a 61-year-old Malay gentleman with underlying hypertension. He was a known case of transglottic carcinoma (T3N0M0), for which he had undergone total laryngectomy, partial thyroidectomy, and selective neck dissection level II to IV. After a laryngectomy, he had a voice prosthesis inserted and was able to acquire prosthetic speech. The patient was using a tracheoesophageal voice prosthesis for three years and was changed every six to eight months until he started to develop complications. At the time of presentation, the patient complained of leaking from the tracheoesophageal puncture site for the past four months. This occurred almost every time he took fluids, especially when drinking large amounts quickly. He also had coughing episodes post-leaking. Fortunately, there was no leaking or choking on taking solid food. Additionally, he was unable to phonate via the prosthesis for the past five months. There were no signs of infection, discolored sputum, fever, chest pain, chesty cough, or shortness of breath. Clinically there was no redness, swelling, or tenderness over the stoma. At the TEF site, the prosthesis was seen, partially embedded in granulation tissue with a small residual fistula seen. The patient was given oral antibiotics (oral amoxycillin clavulanate 15 mg/kg/dose BD) for two weeks and the prosthesis was left in situ in view of the high likelihood of spontaneous esophageal extrusion. Two weeks later, the prosthesis had completely extruded and a small residual fistula was observed, with resolving signs of inflammation. The patient was planned for TEF closure under general anesthesia in view of acceptable anesthetic risks. A horizontal skin incision was made above the tracheal stoma, and a subplatysmal skin flap was raised. The serosal wall of the esophagus and the fistula tract were delineated with sharp dissection. Subsequently, a cuff of mucosa surrounding the tracheal and the esophageal ends of the fistula was removed and refashioned. The esophageal mucosa was repaired vertically, followed by another layer of repair at the serosal layer using polyglactin 3-0 sutures. The tracheal defect was repaired horizontally using polyglactin 3-0 sutures, followed by skin flap closure. Postoperative care included prophylactic antibiotics (oral amoxycillin clavulanate 15 mg/kg/dose BD) for three days, and analgesia (oral paracetamol 10 mg/kg/dose TDS), sterile water dressing, and exclusive nasogastric tube feeding for two weeks. At two weeks clinic visit, evidence of healing was noted, and no leak was observed on a clear fluid swallowing test. The nasogastric tube was removed, and the patient was instructed to resume feeding perorally. No intermediate complications were noted, and the patient started to use the electrolarynx for voice rehabilitation two months after the closure of TEF. Long-term follow-up at three years post-procedure showed good healing and no recurrence of TEF.

Case 3

The third case was of a 69-year-old Chinese male, with underlying hepatitis C, and a history of transglottic carcinoma (T3N0M0) for which he underwent total laryngectomy and total thyroidectomy. He completed adjuvant radiotherapy of 40 Gy, over 20 cycles. Subsequently, the patient underwent secondary TEP and was on voice prosthesis for three years. He had defaulted follow-up and neglected regular cleaning and change of the prosthesis due to significant financial constraints. A review in the clinic showed the prosthesis to be infected, with an abnormally large TEF. Upon removal of the voice prosthesis, it was noted that the prosthesis was fragile, with uncontrolled aspiration of saliva. A nasogastric tube was inserted through the TEF; however, it was not big enough to fully cover the TEF. The patient was kept strictly nil by mouth and was started on total parenteral nutrition. Subsequently, a nasojejunal tube was inserted for enteral feeding. Despite rigorous nutritional optimization over a duration of four weeks, minimal improvements in biochemical parameters were achieved. His serum albumin was 37.9 g/L and hemoglobin was 13 g/dL.

**Figure 1:**
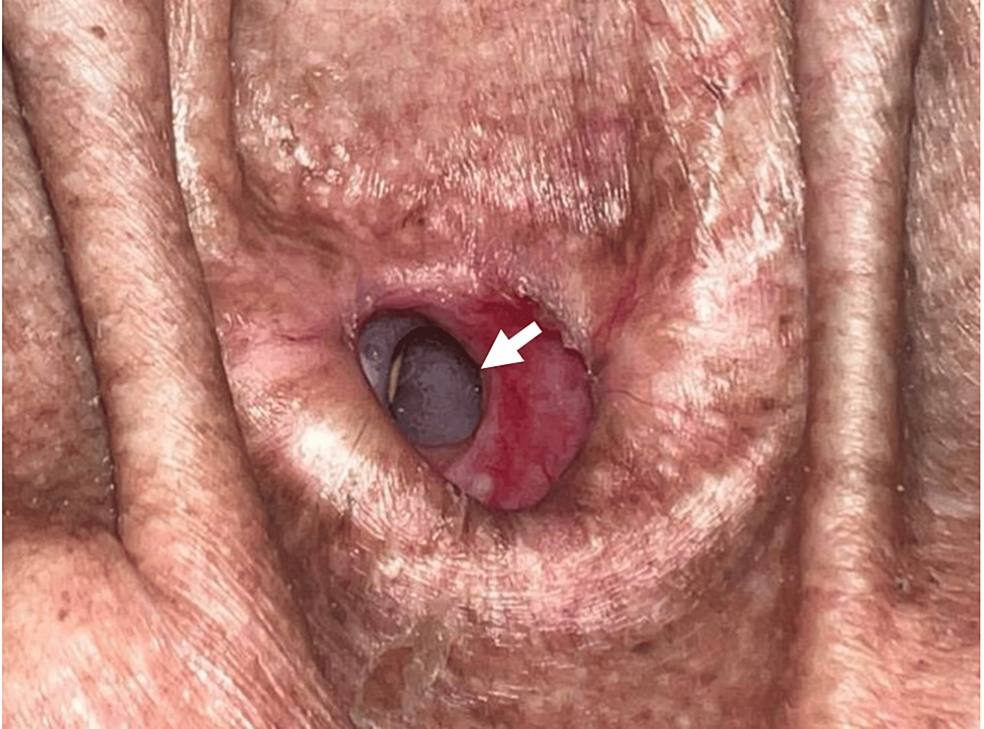
Arrow showing a relatively big tracheoesophageal fistula of 0.9 cm in diameter.

The patient subsequently underwent primary closure of TEF under general anesthesia. Intraoperatively it was found that the TEF size was relatively big at 0.9 cm (Figure 1).

**Figure 2:**
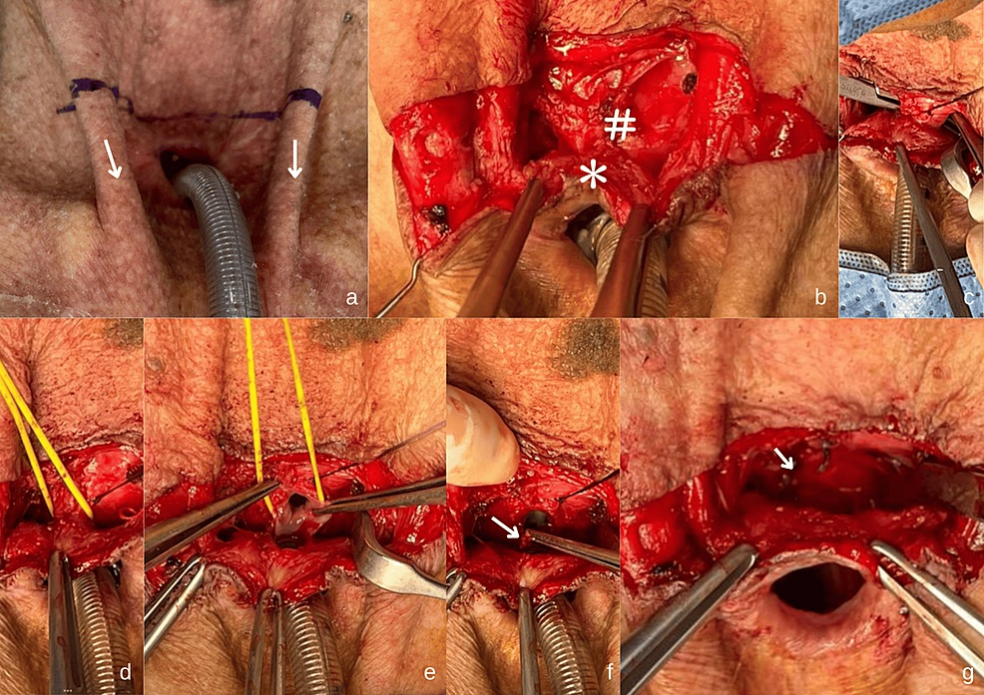
Anterior neck profile showing the surgical steps undertaken. (a) A horizontal skin incision was marked above the tracheoesophageal fistula, extending from the right to the left sternocleidomastoid muscles (white arrow). (b) Subplatysmal flaps were raised superiorly and inferiorly and a plane was developed between the trachea (*) anteriorly and cervical esophagus (#) posteriorly. (c) Identification of the serosal wall of the esophagus and the fistula tract with sharp dissection. (d) Application of vascular loop improves surgical handling during delineation. (e) Separation of posterior tracheal wall and anterior esophageal wall. (f) Refashioning of the epithelial lining (white arrow) at the esophageal side of the fistula. (g) Vertical closure of the esophageal wall (white arrow) and horizontal closure of the tracheal wall with simple interrupted sutures (polyglactin 3-0). Following the two-layer closure of the tracheoesophageal fistula, the skin flap was repositioned and closed using simple interrupted non-absorbable sutures.

A horizontal incision was made above the tracheal stoma, and a subplatysmal skin flap was raised (Figure 2(a),(b)). The serosal wall of the esophagus and the fistula tract were delineated with sharp dissection, isolated, and the TEF was incised in total (Figure 2(c)-(e)). Subsequently, a cuff of mucosa surrounding the tracheal and the esophageal ends of the fistula was removed and refashioned (Figure 2(f)). The esophageal mucosa was repaired vertically, followed by another layer of repair at the serosal layer using polyglactin 3-0 sutures (Figure 2(g)).

The tracheal defect was repaired horizontally using polyglactin 3-0 sutures, followed by skin flap closure. Postoperative care included prophylactic antibiotics (oral amoxycillin clavulanate 15 mg/kg/dose BD) for three days, and analgesia (oral paracetamol 10 mg/kg/dose TDS), sterile water dressing, and exclusive nasojejunal tube feeding for two weeks.

Unfortunately, the patient was non-compliant with the postoperative instructions and started to take orally on postoperative day 5. Subsequently, on postoperative day 21, leakage was noted from the repair site. Exclusive nasojejunal feeding was reemphasized and the patient was discharged with nasojejunal feeding.

**Figure 3:**
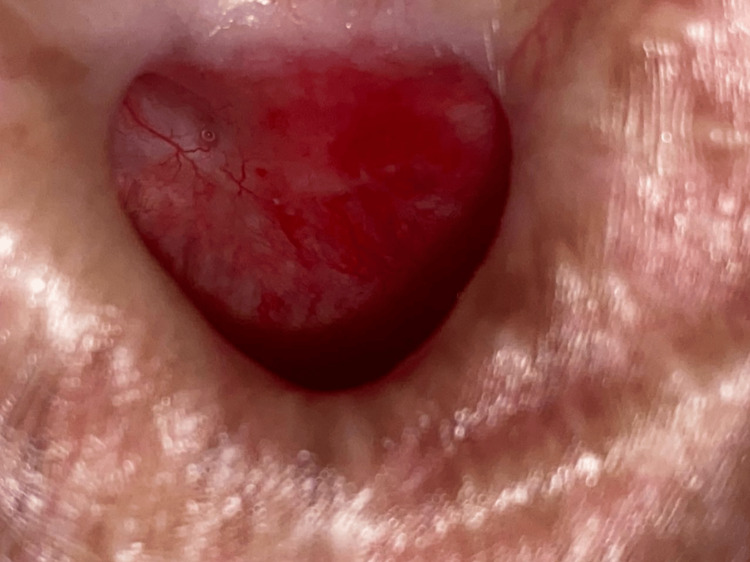
Close-up view of the tracheal stoma showing evidence of complete healing of the repair site in Case 3.

During outpatient clinic follow-up, serial scoring and refashioning were performed over the leakage site to promote wound healing. Finally, at three months postoperatively, there were no more fistulae seen, with evidence of complete healing (Figure 3). Oral feeding was successfully commenced and the nasojejunal tube was removed. Long-term follow-up at one year post-procedure showed good healing and no recurrence of TEF.

## Discussion

Post-laryngectomy voice prosthesis placement is a very important form of voice rehabilitation [3]. Restoration of voice via prosthetic speech occurs in 73% to 78% of cases [5,6] as compared to 5% of patients who attempt to use purely esophageal speech. Intermittent leakage stands out as the most prevalent issue following TEP, with a study indicating that nearly 10% of patients experience leakage of saliva and food from the puncture site [7]. According to another study, leakage occurred in as much as 26% of TEPs [4]. This leakage may be classified into the prosthetic or periprosthetic types.

Prosthetic leakage is due to incompetence of the valve in the prosthesis or incorrect positioning of the TEF. Notably, valve incompetence often results from *Candida albicans* infestation of the silicone material [8]. Negative esophageal pressures are also thought to be a factor for leakage through the valve lumen of the prosthesis [4]. However, newer candida-resistant materials are being incorporated into prosthetic valves to mitigate the need for frequent replacements [4].

The periprosthetic leakage is frequently observed in patients with a significant and persistent TEF widening. This complication was observed in 3.4% of TEP cases [4]. Widening of the TEP contributed toward leakage of fluid and/or food surrounding the tracheo-esophageal prosthesis. It can also lead to displacement and dislodgement of the prosthesis to the esophageal or tracheal lumen. These relatively uncommon complications may arise from fistula wall pressure necrosis from various local and systemic factors [5]. In our case series, the first patient initially experienced successful voice rehabilitation with TEP, with no complications. However, after 11 years, a secondary infection of the prosthesis occurred, and the prosthesis was displaced, although there was no leakage. The second patient developed a secondary infection, leakage, and inability to produce voice from the prosthesis despite being compliant with regular change of the prosthesis. Patient 3 developed a secondary infection after three years of prosthesis use with a history of radiotherapy and malnutrition. Coul et al. found that previous radiotherapy resulted in a shorter speech valve lifetime [9].

While exchanging the prosthesis or its temporary removal typically leads to the shrinking of the fistula, certain cases exhibit continued distention of the fistula despite these interventions. In such instances, closure of the fistula becomes imperative to prevent potentially life-threatening complications such as aspiration pneumonia [3]. Various surgical and non-surgical procedures for TEP closure have been introduced. Conservative non-surgical procedures include the placement of a salivary bypass tube; however, this is not suitable for long-term use [10]. Other techniques involve the use of a silicone button or different biocompatible injections, sling sutures around the TEP mucosa, and silicone rings around the voice prosthesis [10,11].

Surgical closure of TEP, however, is not as straightforward as its creation. Traditional surgical repair involves three-layer closure with the placement of healthy and well-vascularized tissue between the trachea and the esophagus. A two-layer closure without interposition has also been described for smaller fistulas [12]. The interposition tissue used includes a biosynthetic mesh [13], a de-epithelialized deltopectoral flap [14], or dermis tissue. Muscle rotation flaps such as the pectoralis major and sternocleidomastoid muscles have also been recommended, but they may obstruct the lumen of the esophagus and/or the trachea. For larger defects, a radial forearm free flap with vascular anastomosis has been used [15]. Additionally, suture ligation on both ends of the fistula without removing the fistula tract has also been described [9]. In our case, the TEF is cut from both the tracheal and the esophageal ends, and a two-layer closure is done without any interposition. The two layers are the anterior esophageal wall and the posterior tracheal wall. Absorbable sutures are recommended in this region and therefore they were used. However, due to the smaller size of the fistulas, no interpositional flap was placed. However, the technique of closure also depends on tissue conditions within the TEF, tissue conditions of the neck skin, comorbidities, depth and location of the TEF, and prior radiation therapy [16].

## Conclusions

This case series illustrates the diverse presentation of complications in patients with TEP and voice prostheses. The successful closure of fistulas and good postoperative outcomes demonstrate the potential for positive outcomes of a simple two-layer closure even in patients with comorbidities. It is essential for healthcare providers to be aware of the potential complications associated with TEP and to monitor patients closely for signs of complications. Timely intervention, as demonstrated in these cases, can be lifesaving and can significantly improve the quality of life for laryngectomees.
